# Impact of educational level on EDACS performance for chest pain risk stratification: A prospective cross-sectional study

**DOI:** 10.1097/MD.0000000000050672

**Published:** 2026-09-18

**Authors:** Rezan Karaali, Guner Yurtsever

**Affiliations:** a Department of Emergency Medicine, Faculty of Medicine, Izmir Democracy University, Izmir, Türkiye; b Department of Emergency Medicine, Izmir Ataturk Training and Research Hospital, Izmir, Türkiye.

**Keywords:** cardiovascular diseases, chest pain, educational status, emergency department, risk assessment, troponin

## Abstract

The Emergency Department Assessment of Chest Pain Score (EDACS) is a validated tool for identifying patients at very low risk of major adverse cardiac events (MACE), enabling safe discharge without further testing. EDACS incorporates symptom characteristics such as location, pattern, radiation, association with respiration, and cardiovascular risk factors—information typically based on patient self-report. This study aimed to evaluate whether educational level affects patients’ ability to describe symptoms, to evaluate risk refinement between the EDACS and the objectively enhanced EDACS-ADP (Accelerated Diagnostic Pathway) classifications. This prospective, cross-sectional, single-center study included patients presenting to a tertiary emergency department with chest pain. Patients were categorized into 3 groups based on educational level. EDACS and EDACS-ADP were calculated for each patient. Risk reclassification and the influence of educational level were analyzed using McNemar’s test and multivariable logistic regression. Of 4151 patients admitted to the emergency department with complaints of chest pain during a one-month period, 177 were included. 62.7% were male; the median age was 61.0 years. Overall, 38.4% (48/125) of patients in the EDACS-ADP high-risk category were initially classified as low-risk by EDACS (*P* < .001). The reclassification rate was highest in Group 1 (low education) at 42.9%, in Group 2 (38.9%), and Group 3 (33.3%) (*P* < .001 for all). Multivariable logistic regression identified female gender, coronary arterial disease, and smoking as independent predictors of reclassification. Our findings suggest that EDACS-ADP provides additional objective risk refinement across patients with different educational backgrounds.

## 1. Introduction

Chest pain is a common symptom among emergency department (ED) patients, and acute chest pain accounts for 8–10% of all ED visits.^[1]^ There are 7–8 million chest pain visits annually in the United States, approximately 10%–15% of which are acute coronary syndrome (ACS) cases.^[2]^ However, 1–2% of these patients are misdiagnosed and discharged from the ED.^[3,4]^ Missing the diagnosis of ACS can lead to death or other adverse outcomes. Therefore, patients with chest pain in the ED undergo extensive testing to rule out ACS.^[5–7]^ However, only a small fraction of these patients has ACS. Several risk stratification scoring systems have been developed to distinguish these low-risk patients.^[6]^ One of these scores is the Emergency Department Chest Pain Assessment Score (EDACS). The EDACS collects information on age, gender, cardiovascular risk factors, symptoms, and signs, and classifies patients as low and high risk, below and above a reference point of 16, respectively.^[8,9]^ It has been recommended to identify cases with a very low risk of major adverse cardiac events (MACE) and who can be safely discharged from the emergency department for outpatient observation and management only.^[10,11]^ The chest pain location, pattern, spread, relationship to breathing, and cardiovascular risk factors used in calculating the EDACS are evaluated based on information obtained from the patient.^[5,8,11]^ When EDACS is combined with ECG and high-sensitivity cTn results, this score is referred to as the accelerated diagnostic pathway (EDACS-ADP).^[12]^

Studies have shown that the presence of comorbidities in patients presenting to the emergency department with chest pain, as well as limitations in communicating their current medical conditions to physicians, is affected by socioeconomic status.^[13]^ Furthermore, it has been reported that patients with low socioeconomic status are more frequently classified as nonurgent in triage, are discharged in less than 4 hours, and exhibit a higher rate of readmission with complaints of chest pain.^[14]^

Based on these studies, we considered that the parameters used in calculating the EDACS, which heavily rely on patient-reported symptom descriptions, might be associated with the patient’s educational level. In this context, educational level was evaluated not as a direct measure, but as a surrogate indicator of broader social determinants of health, including health literacy, socioeconomic status, and communication ability. In contrast, the EDACS-ADP incorporates objective data, such as ECG findings and troponin levels. Therefore, we hypothesized that the objective diagnostic components of EDACS-ADP would provide additional risk refinement compared to EDACS alone, particularly among patients with diverse educational backgrounds where variations in surrogate indicators like health literacy may influence subjective symptom reporting.

## 2. Methods

The study was planned as a single-center, prospective, cross-sectional study. Our hospital is a tertiary care academic medical center, and our daily patient count is 1700–2000 in 24 hours. Our emergency department is the city’s largest emergency department with emergency medicine specialists, emergency physician assistants and emergency certified nurses, providing service 24h/7d.

Patients aged 18 years or older who presented to the emergency department with chest pain compatible with acute coronary syndrome as the primary symptom, had stable and relatively normal vital signs not requiring emergency intervention, and had a high-sensitivity cardiac Troponin T (hs-cTnT) test requested were included. Those who did not speak Turkish or could not communicate due to conditions such as dementia, had ST-segment elevation myocardial infarction (STEMI), were under 18 years old, had post-traumatic chest pain, experienced cardiopulmonary arrest, did not consent to participate or could not be followed up, lacked both 0-hour and 1-hour hs-cTnT results, or had missing data from parameters used in the EDACS-ADP calculation were excluded. The EDACS was calculated using the patients’ age, gender, presence of comorbidities (coronary artery disease, diabetes, hypertension, smoking), and nature of chest pain (radiation, relationship with breathing, increase/decrease with palpation, presence of diaphoresis). The EDACS calculation is based on 7 quick questions, each with a specific score; the maximum score that can be obtained is 34 and the minimum score is 10. EDACS defines 2 levels according to the occurrence of MACE: low risk (<16 points) and not low risk (≥16 points).^[5,8]^ Chest pain was assessed as specified in the 2020 European Society of Cardiology (ESC) Guidelines for the management of acute coronary syndromes in patients without persistent ST-segment elevation. Typical chest discomfort is characterized by a retrosternal sensation of pain, pressure, or heaviness (“angina”) radiating to the left arm, both arms, the right arm, the neck, or the jaw, which maybe intermittent (usually lasting several minutes) or persistent. Additional symptoms such as sweating, nausea, epigastric pain, dyspnea, and syncope may be present. noncardiac pain includes isolated epigastric pain, indigestion-like symptoms, and isolated dyspnea or fatigue.^[15]^

Cardiovascular disease risk factors used in calculating the EDACS were defined as known coronary artery disease (CAD), previous acute myocardial infarction (AMI), and any coronary revascularization including percutaneous coronary intervention (PCI) and coronary artery bypass graft surgery (CABG). The listed risk factors included diabetes mellitus, hypertension, hyperlipidemia, current smoking habit, and any family history of CAD at an an early age. According to the standard practice of our institution, troponin levels are requested for patients with suspected coronary artery disease at the time of admission to the emergency department (0th hour) and one hour later (1st hour). Troponin Ths values obtained from patients at 0 and 1 hours were measured for the EDACS-ADP. Cardiac troponin Ths was studied on the ROCHE HITACHI Cobas 8000 device. The normal value of troponin is < 14 ng/L. Troponin levels above this value were considered positive. New ischemic changes in the Electrocardiogram (ECG) obtained from the patients were defined according to the following criteria: New ischemic changes in the ECG were defined as ST segment depression of at least 0.05 mV in 2 adjacent leads, T wave inversion of at least 0.1 mV, or Q waves of at least 0.1 mV depth and 30 ms width in 2 adjacent leads. The EDACS-ADP identifies patients appropriate for early discharge if they have an EDACS < 16, no new ischemia on ECG, and negative serial troponin results at 0 and 1 hours after ED arrival. Patients who met any of these criteria were classified as high risk according to EDACS-ADP.^[5,8,11]^ To minimize potential sources of bias, all eligible patients presenting with chest pain to the tertiary care emergency department during this study were consecutively enrolled on randomly selected days, regardless of triage level or initial clinical suspicion of acute coronary syndrome (ACS). Initial patient symptom assessments and clinical histories were obtained directly through face-to-face interviews conducted at the time of presentation by the study investigators, who are emergency medicine specialists, using a standardized clinical checklist. While the use of a standardized checklist ensured methodological consistency across evaluations, a formal statistical analysis of interobserver variability among the investigators was not performed. Furthermore, to prevent potential observer bias, EDACS was assigned based on the standardized checklist prior to and independently of the review of final electrocardiogram (ECG) interpretations and troponin laboratory results. ECG interpretations were subsequently conducted independently by emergency medicine specialists using predefined criteria to preserve consistency in risk assessment procedures.

In Turkey, the formal education system consists of 4 years of primary school, 4 years of middle school, and 4 years of high school education. Following high school, students take a national university entrance examination. Based on the results, they may be admitted to a 2-year associate degree program or a 4-year undergraduate (bachelor’s) program. Postgraduate education, including master’s and doctoral degrees, is available following completion of undergraduate studies.^[16]^ Patients were divided into 3 groups based on their educational levels. While forming the groups, the last completed level of education was considered for patients who were no longer enrolled in school, and the current level of enrollment was used for those still pursuing their education. Group 1 (illiterate, literate, primary school), group 2 (secondary school, high school, associate degree), group 3 (undergraduate, university graduate, doctorate, academician). The obtained data were compared among these 3 groups. To enhance statistical power and prevent model instability during multivariable analysis due to small subgroup sizes, patient educational levels were regrouped in alignment with the structural dynamics of the national education system. In this context, associate degree graduates were combined with secondary and high school graduates into a single category. The rationale for this consolidation was twofold: first, the proportion of associate degree graduates within the study cohort was independently insufficient to form a distinct statistical group; second, within the national educational framework, associate degree programs are primarily vocational and technical in nature, focusing on practical skills rather than comprehensive academic and health-related curricula. Therefore, in terms of broader social determinants of health and health literacy patterns, this subgroup aligns more closely with secondary and high school education levels than with advanced four-year baccalaureate or postgraduate academic degrees.

The study protocol was approved by the Institutional Review Board and Ethics Committee of Buca Seyfi Demirsoy Training and Research Hospital (Approval Date: 28.02.2024, Decision No: 239) The study was conducted in strict accordance with the ethical principles outlined in the Declaration of Helsinki. Prior to enrollment, the study investigators provided all eligible patients with a detailed explanation of the study objectives, and written informed consent was obtained from all individual participants included in the study.

### 2.1. Statistical method

Statistical analyses were performed using R software (version 2024.09.0 + 375) in the study. The Shapiro–Wilk test was employed to assess the conformity of continuous variables to a normal distribution. When presenting descriptive statistics, mean ± standard deviation values were given for normally distributed data, and median (1st quartile [Q1]–3rd quartile [Q3]) values were given for non-normally distributed data. The McNemar test was used to assess the agreement between the 2 measurements. This test was applied to determine whether the transitions between low and high-risk categories between EDACS and EDACS-ADP were significant. “dplyr” and “stats” packages were used. Statistical significance level was accepted as *P* < .05.

To evaluate the factors influencing the reclassification from low to high risk between EDACS and EDACS-ADP based on educational level, a multivariable logistic regression analysis was performed. The regression model included potential confounding variables such as age, sex, coronary artery disease history, dyslipidemia, diabetes, hypertension, smoking status, and family history. Reference categories for each variable were determined based on clinical relevance and the distribution of data.

The sample size was calculated in the R program for the McNemar test, aiming to reach approximately 64 people when the significance level (α) was taken as 0.05, statistical power (1−β) as 0.95, p1 = 0.6 (expected proportion of individuals remaining in the EDACS low-risk category), and p2 = 0.3 (expected proportion of individuals moving from low risk to high risk with EDACS-ADP).

## 3. Results

During the one-month period in which the research was conducted, a total of 4151 patients presented to our emergency department with chest pain. Of these patients, 121 (2.9%) were diagnosed with STEMI, 208 (5%) with decompensated heart failure, 862 (20.76%) with chest pain following trauma, and 2466 (59.4%) with noncardiac chest pain. 312 (7.52%) patients either did not provide consent or sufficient data could not be obtained. A total of 177 patients who met the study criteria and provided informed consent were included in the study. The median age of our patients was 61.0 [49.0; 71.0] years, and 62.7% were male. 51.4% of the patients had a history of CAD, 23.2% had hyperlipidemia, 31.6% had DM, and 62.7% had HT. Ischemic findings were detected in 43.5% of the ECGs obtained at the time of admission to the emergency department. When the parameters used in calculating the EDACS were examined, pain radiating to the left jaw was seen in 43.2%, and pain worsening with breathing in 41.8%. The median troponin value was found to be 7.24 [3.20; 24.5] ng/L (normal range is 14 ng/L). Based on educational status, the highest concentration of patients was observed in Group 2 (39.5%). When the outcomes of the patients were examined, it was determined that 63.3% underwent coronary arterial angiography. According to the EDACS evaluation, 43.5% of patients had a score ≥ 16 and were categorized as high risk. When EDACS‐ADP, which incorporates ECG and troponin results, was applied, 70.6% of patients were classified as high risk (Table 1). Mean EDACS for Groups 1–3 were found to be 13.0 ± 6.55, 14.3 ± 6.98, and 13.9 ± 7.75, respectively. While EDACS identified low-risk rates as 66.7%, 52.9%, and 48.9% respectively, with EDACS-ADP, the low-risk rates were found to be 41.7% in Group 1, 22.9% in Group 2, and 23.4% in Group 3, respectively (Table 2).

**Table 1:** Baseline demographic characteristics, clinical parameters, and risk classification of the study population.

Variables	*n* (%)	
Age, median [Q1;Q3]	61.0 [49.0;71.0]	
Gender		
Male	111 (62.7)	
Female	66 (37.3)	
Diaphoresis	64 (36.2)	
Pain radiates to arm, shoulder, neck, or jaw	76 (43.2)	
Pain is reproduced by palpation	26 (14.7)	
Pain occurred or worsened with inspiration	74 (41.8)	
CAD	91 (51.4)	
Dyslipidemia	41 (23.2)	
DM	56 (31.6)	
HT	111 (62.7)	
Smoking	74 (42.0)	
Family history	38 (21.5)	
New ischemic ECG changes	77 (43.5)	
Troponin, median [Q1;Q3] ng/L	7.24 [3.20;24.5]	
EDACS, mean ± SD	13.7 ± 7.04	
Education level		
Group 1	60 (33.9%)	
Group 2	70 (39.5%)	
Group 3	47 (26.6%)	
Outcome		
Coronary angiography	Emergency angiography	102 (91.1)
	Coronary ICU	4 (3.57)
	Cardiology ward	6 (5.36)
Discharge from ED	65 (36.7)	
Troponin results		
<14 ng/L	106 (59.9)	
≥14 ng/L	71 (40.1)	
EDACS		
Low risk	100 (56.5)	
High risk	77 (43.5)	
EDACS-ADP		
Low risk	52 (29.4)	
High risk	125 (70.6)	

Data are presented as numbers (percentages) or means ± standard deviations as appropriate.

Group 1 (illiterate, literate, primary school), group 2 (secondary school, high school, associate degree), group 3 (undergraduate, university graduate, doctorate, academician).

CAD = coronary artery disease, DM = diabetes mellitus, EDACS = Emergency Department Assessment of Chest pain Score, EDACS-ADP = Emergency Department Assessment of Chest pain Score- Accelerated Diagnostic Pathway, HT = hypertension.

**Table 2:** Comparison of clinical score values and risk stratifications across patient educational attainment groups.

	Group 1	Group 2	Group 3
	*n* = 60	*n* = 70	*n* = 47
EDACS, mean ± SD	13.0 ± 6.55	14.3 ± 6.98	13.9 ± 7.75
EDACS, n(%)			
Low risk	40 (66.7%)	37 (52.9%)	23 (48.9%)
High risk	20 (33.3%)	33 (47.1%)	24 (51.1%)
EDACS-ADP, n(%)			
Low risk	25 (41.7%)	16 (22.9%)	11 (23.4%)
High risk	35 (58.3%)	54 (77.1%)	36 (76.6%)

Continuous data are presented as means ± standard deviations (SD), and categorical data are expressed as frequencies with corresponding percentages [n (%)]. Group 1, Group 2, and Group 3 represent the distinct educational strata of the study population. Group 1: illiterate, literate, primary school, Group 2: secondary school, high school, associate degree, Group 3: undergraduate, university graduate, doctorate, academician.

EDACS = Emergency Department Assessment of Chest pain Score, EDACS-ADP = Emergency Department Assessment of Chest pain Score- Accelerated Diagnostic Pathway.

**Figure 1.**
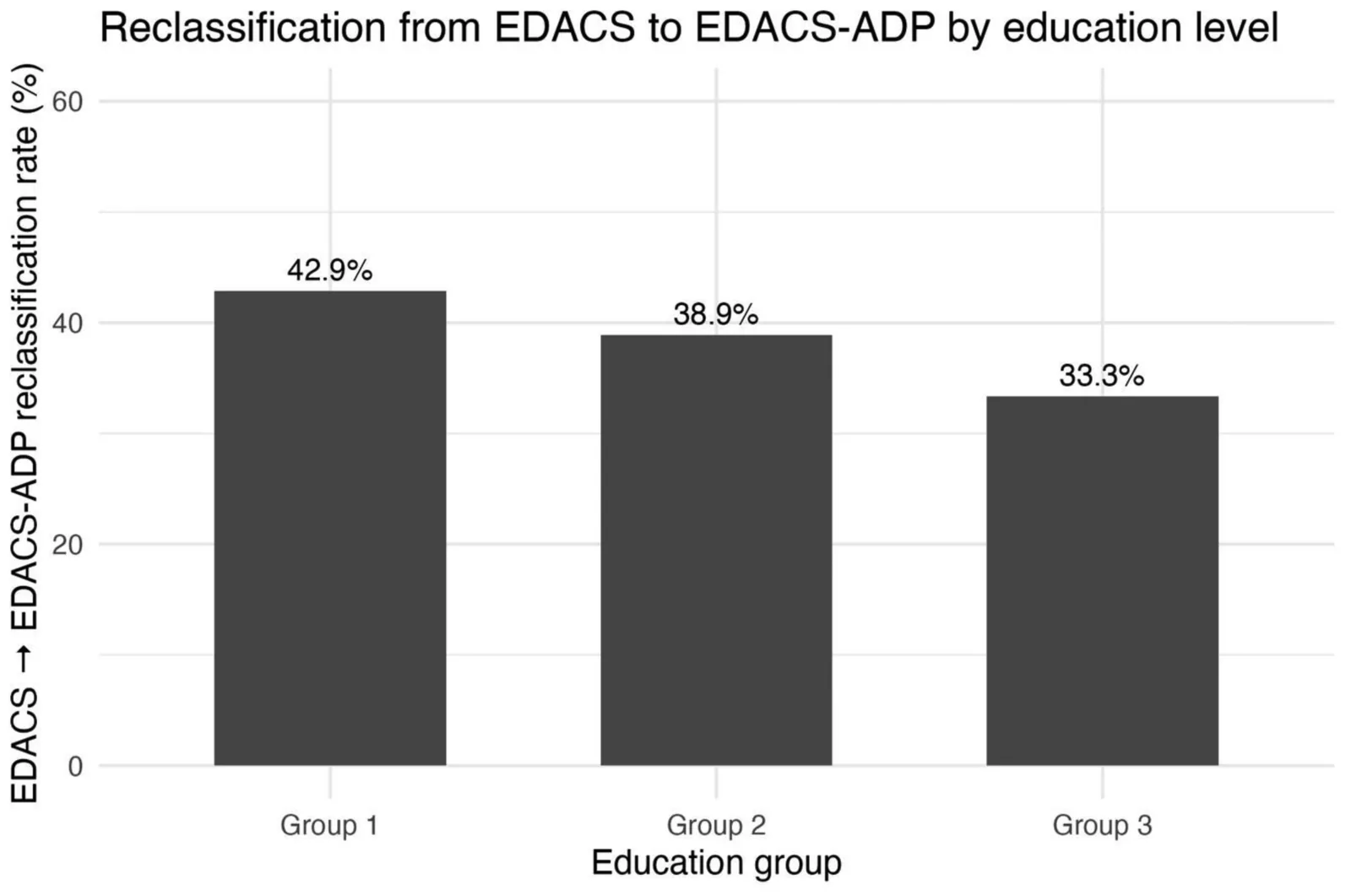
Patient risk reclassification rates across different educational groups. The bar chart illustrates the percentage of patients initially categorized as low risk who were subsequently reclassified into the high-risk category. Group 1, Group 2, and Group 3 denote the distinct educational attainment strata of the study cohort. Risk reclassification reflects the transition from EDACS to EDACS-ADP. Vertical axes represent the reclassification rates expressed as percentages (%). EDACS = Emergency Department Chest Pain Score, EDACS-ADP = Emergency Department Chest Pain Score-Accelerated Diagnostic Pathway. Group 1: illiterate, literate, primary school, Group 2: secondary school, high school, associate degree, Group 3: undergraduate, university graduate, doctorate, academician.

We further evaluated the reclassification of patients initially identified as low risk by EDACS but who were shifted to the high-risk category by EDACS-ADP. Within the entire patient cohort, 38.4% (48/125) of those in the EDACS-ADP high-risk category were found to have been initially classified as low risk by EDACS. This reclassification was determined to be statistically significant (*P* < .001, χ^2^ = 48.0). The analysis of groups categorized by educational level revealed that the transition rates from low risk in EDACS to high risk in EDACS-ADP varied across groups. The highest transition rate from low to high risk was observed in Group 1 patients. In this group, 42.9% (15/35) of the EDACS-ADP high-risk patients were initially deemed low risk by EDACS (*P* < .001, χ^2^ = 13.0). For Group 2, 38.9% (21/54) of patients in the EDACS-ADP high-risk category had been originally classified as low risk by EDACS (*P* < .001, χ^2^ = 19.0). This rate was identified as 33.3% (12/36) in Group 3, and the reclassification was also found to be statistically significant (*P* = .001, χ^2^ = 10.0) (Table 3) (Fig. 1).

**Table 3:** McNemar test analysis of risk classification transitions across patient educational strata.

EDACS		EDACS-ADP		p	X^2^
		Low risk	High risk		
All patients	Low risk	52 (100.0%)	48 (38.4%)	<0.001	46.0
	High risk	0 (0.0%)	77 (61.6%)		
Group 1	Low risk	25 (100.0%)	15 (42.9%)	<0.001	13.0
	High risk	0 (0.0%)	20 (57.1%)		
Group 2	Low risk	16 (100.0%)	21 (38.9%)	<0.001	19.0
	High risk	0 (0.0%)	33 (61.1%)		
Group 3	Low risk	11 (100.0%)*	12 (33.3%)‡	0.001	10.0
	High risk	0 (0.0%)**	24 (66.7%)†		

The McNemar test was utilized to evaluate the systematic shifts and proportions of changes in risk assessment when transitioning from the baseline clinical score to the accelerated diagnostic pathway.

Educational strata represent the distinct education levels of the patient cohort. Group 1: illiterate, literate, primary school, Group 2: secondary school, high school, associate degree, Group 3: undergraduate, university graduate, doctorate, academician.

EDACS = Emergency Department Assessment of Chest pain Score, EDACS-ADP = Emergency Department Assessment of Chest pain Score- Accelerated Diagnostic Pathway.

* .

† ;Concordance pairs.

‡ .

** ; Discordance pairs.

A multivariable logistic regression analysis was performed to identify independent predictors of reclassification from EDACS low risk to EDACS-ADP high risk. The model adjusted for age, gender, educational level, history of CAD, dyslipidemia, DM, HT, and smoking status. The analysis demonstrated that female gender and a history of CAD were strong independent predictors, significantly increasing the likelihood of reclassification; females had 2.75-fold higher odds (OR = 2.75, 95% CI: 1.29–5.86, *P* = .009), and patients with CAD had nearly 3-fold higher odds (OR = 2.95, 95% CI: 1.12–7.75, *P* = .028) of being reclassified. Conversely, smoking status and increasing age showed inverse relationships with reclassification; smokers had 60% lower odds of reclassification (OR = 0.40, 95% CI: 0.18–0.88, *P* = .021), and each additional year of age was associated with a slight decrease in reclassification probability (OR = 0.97, 95% CI: 0.94–0.99, *P* = .028). Educational level was not found to be an independent predictor for reclassification, showing an effect size close to baseline (OR = 1.09, 95% CI: 0.69–1.71, *P* = .709). Similarly, no significant relationships were observed for dyslipidemia (OR = 0.56, 95% CI: 0.21–1.51), DM (OR = 0.66, 95% CI: 0.27–1.64), or HT (OR = 1.00, 95% CI: 0.39–2.54), with all confidence intervals crossing 1.0 and *P* > .05 (Table 4).

**Table 4:** Multivariable logistic regression analysis of factors associated with patient risk reclassification.

Variables	β (Estimate)	Std. Error	OR	%95 CI	z value	*P*
Intercept	0.454	0.887	1.57	0.28–8.84	0.512	.609
Education level	0.087	0.232	1.09	0.69–1.71	0.374	.709
Age	−0.034	0.016	0.97	0.94–0.99	−2.196	.028
Gender (female)	1.012	0.387	2.75	1.29–5.86	2.616	.009
CAD	1.080	0.492	2.95	1.12–7.75	2.194	.028
Dyslipidemia	−0.573	0.508	0.56	0.21–1.51	−1.129	.259
DM	−0.408	0.457	0.66	0.27–1.64	−0.893	.372
HT	−0.001	0.483	1.00	0.39–2.54	-0.002	.999
Smoking	−0.917	0.399	0.40	0.18–0.88	-2.301	.021

The multivariable logistic regression model evaluated the factors associated with patient reclassification from the baseline low-risk category into the high-risk group. The model adjusted for potential clinical and demographic confounding variables, including age, sex, hypertension, smoking status, and educational level.

CAD = coronary artery disease, DM = diabetes mellitus, HT = hypertension.

## 4. Discussion

Evaluation of patients with chest pain or symptoms consistent with acute coronary syndrome (ACS) often requires multiple cardiac tests and extended follow-up periods.^[17]^ Identifying low-risk patients can prevent unnecessary testing and prolonged follow-up in the emergency department. Thus, high-risk patients can undergo more extensive testing and receive more dedicated time for evaluation.^[5,10]^ The primary goal for emergency physicians should be to identify low-risk patients. EDACS, which was developed for this purpose, has become a preferred risk scoring system for identifying low-risk patients for MACE in patients presenting with chest pain. In past studies, the pooled NPV for 30-day MACE was found to be 99.23% and 99.94%.^[9,13]^

We found that 38.4% of patients classified as low risk according to EDACS were classified as high risk according to EDACS-ADP. Similar results were reported by Nilsson et al (2021), who found that EDACS-ADP reclassified some patients initially considered low risk by EDACS into the high-risk group.^[7]^ Yoo et al (2024) have compared EDACS, troponin + ECG, and EDACS-ADP, consistently reporting that EDACS-ADP demonstrates the highest sensitivity and specificity in identifying low-risk patients.^[4]^ Similarly, Zaboli et al (2021) emphasized that EDACS alone, without troponin levels, may misclassify patients, underlining the complexity of chest pain evaluation.^[18]^ However, none of these studies clearly explained the reason behind this reclassification. Although binary analysis (McNemar test) demonstrated a significant association between educational level and risk reclassification, this relationship lost statistical significance in the multivariable model after adjustment for age, sex, hypertension, and smoking. The multivariable analysis explicitly identified educational level as not being an independent predictor of reclassification after adjustment (OR = 1.09, 95% CI: 0.69–1.71, *P* = .709). These findings indicate that educational level does not exert a direct causal influence on symptom reporting or EDACS performance but rather functions as a surrogate marker for broader social determinants of health, encompassing health literacy, socioeconomic status, and communication ability. In this context, formal education likely serves as an indirect indicator reflecting a patient’s familiarity with medical concepts and health-related communication, which can be closely interrelated with demographic variables such as age and sex. For instance, Mestral et al (2017) observed that individuals within lower educational strata may exhibit limited awareness of their specific cardiovascular risk factors or medical history, potentially leading to varied symptom descriptions during clinical encounters due to limited health literacy and unfamiliarity with medical terminology.^[19]^ Furthermore, from an epidemiological and socioeconomic perspective, educational attainment is strongly linked to health disparities. This is supported by Hahad et al (2024), whose meta-analysis demonstrated that socioeconomic deprivation and lower educational attainment are tied to heightened cardiovascular risk,^[20]^ as well as Kimenai et al (2022), who showed that incorporating socioeconomic indicators into established cardiovascular risk scores improves predictive performance in disadvantaged populations.^[21]^ Therefore, the statistically significant differences observed across educational groups in our unadjusted analyses are likely mediated through these complex, interrelated socioeconomic and health literacy pathways rather than representing an isolated or direct causal effect of education itself.

Consequently, objective diagnostic components provide additional risk refinement across patients with different educational backgrounds, while educational level itself was not an an independent predictor after multivariable adjustment. The role of education in risk classification should be interpreted in the context of other contributing variables. Future risk models may benefit from integrating these interacting factors to support more equitable and accurate clinical decision-making.

## 5. Limitation

This study has several limitations. First, it was conducted in a tertiary care emergency department, which may affect the generalizability of the results. Although the overall sample size was adequate, subgroup sizes – particularly across educational levels – were limited, potentially reducing statistical power. Additionally, although patient symptom characteristics were collected prospectively by emergency medicine specialists using a standardized clinical checklist, a formal statistical evaluation of interobserver variability among the study investigators was not performed. Furthermore, while multivariable analysis was used to adjust for key confounders, not all potential confounding variables such as health literacy, cognitive function, or detailed socioeconomic status could be accounted for, introducing the risk of residual confounding. Multiple subgroup comparisons were made without correction for multiple testing; however, these analyses were exploratory in nature. Moreover, the EDACS partially relies on patient-reported symptoms, which may be subject to recall or reporting bias. Finally, the cross-sectional design of the study limits any causal interpretation between educational level and discrepancies in risk classification.

## 6. Conclusion

In conclusion, our findings highlight that EDACS-ADP significantly refines and improves risk stratification for chest pain in the emergency department, providing valuable additional risk assessment across patients with different educational backgrounds. While educational level serves as a surrogate marker for broader social determinants of health – such as health literacy, socioeconomic status, and communication ability – the multivariable analysis demonstrated that educational level itself is not an independent predictor of risk reclassification after adjustment. Instead, the observed associations in unadjusted analyses are likely mediated through complex interactions with clinical and demographic factors, including female gender, age, and a history of CAD. Therefore, the integration of objective diagnostic components in EDACS-ADP effectively addresses variations in subjective symptom reporting without implying a direct causal influence of formal education on clinical score performance.

## Acknowledgments

We would like to thank Emine Kilinc, MD for her contributions and assistance in the data collection process.

## Author contributions

**Conceptualization:** Rezan Karaali.

**Methodology:** Rezan Karaali.

**Resources:** Rezan Karaali, Guner Yurtsever.

**Writing – original draft:** Rezan Karaali, Guner Yurtsever.

**Writing – review & editing:** Rezan Karaali, Guner Yurtsever.
